# Above-Room-Temperature Ferromagnetism in Large-Scale Epitaxial Fe_3_GaTe_2_/Graphene van der Waals Heterostructures

**DOI:** 10.1021/acsnano.5c07732

**Published:** 2025-10-21

**Authors:** Tauqir Shinwari, Kacho Imtiyaz Ali Khan, Hua Lv, Atekelte Abebe Kassa, Frans Munnik, Simon Josephy, Achim Trampert, Victor Ukleev, Chen Luo, Florin Radu, Jens Herfort, Michael Hanke, Joao Marcelo Jordao Lopes

**Affiliations:** † Paul-Drude-Institut für Festkörperelektronik Leibniz-Institut im Forschungsverbund Berlin e.V., Berlin 10117, Germany; ‡ Helmholtz-Zentrum Dresden-Rossendorf, Institute of Ion Beam Physics and Materials Research, Bautzner Landstrasse 400, Dresden 01328, Germany; § QZabre AG, Neunbrunnenstrasse 50, Zürich 8050, Switzerland; ∥ 28340 Helmholtz Zentrum Berlin for Materialien und Energie, Albert-Einstein Straße 15, Berlin 12489, Germany

**Keywords:** MBE, graphene, Fe_3_GaTe_2_, 2D magnets, magnetic materials, spectroscopy, spintronics

## Abstract

Fe_3_GaTe_2_ (FGaT), a two-dimensional (2D) layered ferromagnetic metal, exhibits a high Curie temperature (*T*_C_) of ∼360 K along with strong perpendicular magnetic anisotropy (PMA), making it a promising material candidate for next-generation energy-efficient magnetic devices. However, the vast majority of studies on FGaT to date have been limited to millimeter-sized bulk crystals and exfoliated flakes, which are unsuitable for practical applications and integration into device processing. Also, its combination with other 2D materials to form van der Waals (vdW) heterostructures has only been achieved by flake stacking. Consequently, the controlled large-area growth of FGaT and related heterostructures remains largely unexplored. In this work, we demonstrate the high-quality, large-area growth of epitaxial FGaT thin films on single-crystalline graphene/SiC templates using molecular beam epitaxy. Structural characterization confirms the high crystalline quality of the continuous FGaT/graphene vdW heterostructures. Temperature-dependent magnetization and anomalous Hall measurements reveal robust PMA with an enhanced *T*_C_ well above room temperature, reaching up to 400 K. Furthermore, X-ray absorption and X-ray magnetic circular dichroism spectra provide insight into the spin and orbital magnetic moment contributions, further validating the high *T*_C_ and robust PMA. These findings are highly significant for the future development of high-performance spintronic devices based on 2D heterostructures, with potential applications in next-generation data storage, logic processing, and quantum technologies.

## Graphical abstract

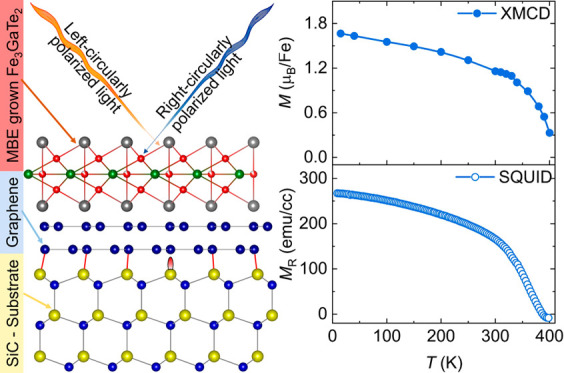

## Introduction

The emergence of two-dimensional (2D) van der Waals (vdW) magnetic materials has opened new frontiers in fundamental physics, in particular in the exploration of exotic magnetic phenomena such as topologically nontrivial spin textures in the 2D limit.^1,2^ They have also been shown to be promising building blocks for the realization of novel devices for spintronics and quantum technologies with low-power consumption.^3,4^ Here, a particularly promising approach is to combine such layered magnets with other 2D crystals (e.g., graphene and WSe_2_) to create vdW heterostructures with multiple functionalities and properties that can be tailored via heterostructure design and proximity-induced phenomena.^5,6^ Moreover, 2D magnetic heterostructures are advantageous over conventional metallic magnetic heterostructures,^7–12^ as their ultrathin nature is expected to facilitate the realization of ultracompact devices for efficient charge and spin transport.^13–17^ In the last couple of years, the library of 2D vdW magnets has expanded rapidly.^18^ Several (anti)­ferromagnetic materials possessing a semiconductor or metallic nature have been identified.^13^ However, only a few of them (typically ferromagnetic metals) exhibit transition temperatures around or above room temperature,^19–21^ which is essential for their implementation in a wide range of applications. Among these materials, the 2D ferromagnetic metal Fe_3_GaTe_2_ (FGaT) has recently gained significant attention due to its exceptional magnetic properties. In addition to having one of the highest reported Curie temperatures (between 350 and 380 K),^20,22^ FGaT also possesses a strong perpendicular magnetic anisotropy (PMA), which is crucial for applications in magnetic memories,^3,23,24^ sensors,^25,26^ and skyrmion-based logic devices.^27,28^

FGaT, which is a close analogue to the vdW ferromagnet Fe_3_GeTe_2_ (FGT), shows a hexagonal symmetry with a space group *P*6_3_/*mmc* (No. 194) and bulk lattice parameters *a* = *b* = 4.09 Å and *c* = 16.07 Å.^22^ The atomic structure of an FGaT single layer consists of a Fe_3_Ga slab, which is sandwiched between two Te slabs [Figure 1a,b]. Adjacent layers are held together by weak vdW forces [Figure 1d], which allows the exfoliation of micrometer-sized thin flakes from bulk single crystals.^20,23,29,30^ Both bulk crystals and flakes have been extremely important for studying the intrinsic properties of FGaT, either alone^20,31,32^ or in combination with other 2D materials via assembly of vdW heterostructures.^25,33–35^ However, they are inherently incompatible with device fabrication processes. Hence, to implement FGaT and related heterostructures into future applications, it is necessary to develop large-area growth of FGaT as crystalline thin films with properties comparable to or even superior to those reported for state-of-the-art bulk crystals and flakes. Furthermore, the ability to synthesize FGaT directly on another functional 2D material, ideally without the use of layer transfer, is also highly demanded to enable large-area vdW heterostructures with ultraclean interfaces, which is critical for the future realization of novel devices where interface-related proximity effects will play a critical role.^36,37^

**Figure 1:**
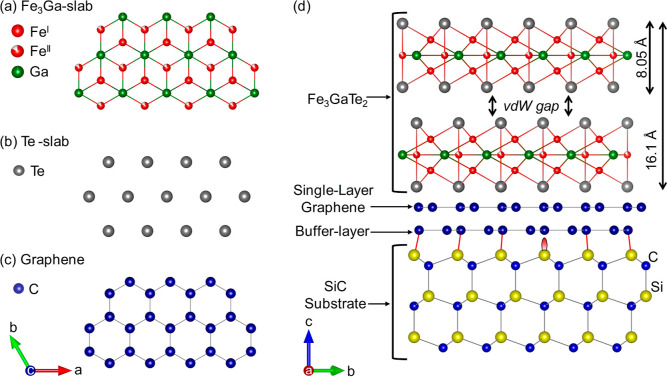
Schematics of the structural configuration of the synthesized FGaT/graphene/SiC(00.1) system.^39^ (a) Top view of the inner Fe_3_Ga slab, where the hexagonal arrangement can be clearly observed. (b) Configuration of the Te atoms forming the outer slabs which encapsulate Fe_3_Ga. (c) Atomic structure of a graphene monolayer. (d) Side view along the [2–1.0] crystallographic orientation of the FGaT layer, which is grown on top of the epitaxial graphene/SiC(00.1) template. Note that the latter also contains a carbon buffer layer at the interface between graphene and SiC, which is covalently bonded to SiC. The axis labels shown in the bottom left and right are related to (a–c) and (d), respectively.

In this work, we demonstrate the large-scale growth of FGaT epitaxial thin films directly on single-crystalline graphene [on SiC(00.1)], see Figure 1c,d, via molecular beam epitaxy (MBE). Continuous and uniform FGaT/graphene vdW heterostructures exhibiting sharp vdW interfaces could be realized, which is a significant advancement with respect to what can be achieved via conventional flake stacking. Importantly, magnetization and magneto-transport characterization performed with different methods reveal the FGaT films on graphene to display robust PMA and high *T*_C_ values approaching 400 K. These results represent a significant step toward the development of ultracompact, all 2D spintronic devices, such as spin valves^36,38^ and spin demultiplexers,^5^ which can be fabricated using vdW heterostructures formed of FGaT and graphene and operate at room temperature.

## Results and Discussion

### Structural Properties

MBE was utilized to realize a high-quality growth of FGaT films with thicknesses of 6, 10, and 32 nm on graphene/SiC(00.1) templates, which were prepared by SiC surface graphitization.^40^ Rutherford backscattering (RBS) spectrometry (see Figure S1 in the Supporting Information) revealed the FGaT films to exhibit an average chemical composition close to Fe_3.15_GaTe_1.47_, namely, Fe_3+*x*_GaTe_2–*y*_. The FGaT growth process was continuously monitored by in situ RHEED. The bottom and upper panels in Figure 2a correspond to the RHEED patterns of the graphene/SiC(00.1) template and of a ∼10 nm thick FGaT film, respectively. The RHEED pattern of the FGaT film shows sharp and continuous streaks, confirming the successful epitaxial growth of an FGaT 2D film having a smooth surface. Based on the position of the streaks, we could extract the in-plane lattice parameters of the FGaT film to be *a* ∼4.08 Å. The in-plane structural properties were investigated in more detail by utilizing synchrotron-based grazing incidence diffraction (GID) measurements. This measurement allows us to extend the areas in reciprocal space and provide a comprehensive view of the existing and diffracting in-plane lattice planes and their azimuthal distribution, as shown in Figure 2b–d. One particular feature of this technique refers to the fact that the diffraction vector contains exclusively in-plane components (i.e., the vertical component practically equals zero because of the grazing incidence and exit). In other words, GID approaches the ideal case of a noncoplanar geometry where the surface normal **n** appears perpendicular to the scattering plane. Consequently, a 2D GID reciprocal space map, as in Figure 2b, shows the scattered X-ray intensity parallel to the sample surface. It can be seen as a compiled sequence of concentric arcs (around the reciprocal origin and probing along *q*_angular_) with an increasing diameter, given by *q*_radial_. In addition to well-defined reflections from the SiC substrate (white circles), there are other contributions due to the FGaT layer. Most of them show a particular in-plane arrangement with respect to the substrate; e.g., following the arc due to FGaT{22.0} net planes, one can see pronounced maxima every 60° and weaker ones in between [Figure 2c]. This shows a preferential arrangement in which FGaT[11.0] is parallel to SiC[10.0] and complementary FGaT[10.0]∥SiC[11.0]. The areas below the red (D1) and green (D2) curve fittings in Figure 2c give a ratio of about 4:1 for the bimodal domain distribution. Figure 2d shows a line plot in the radial direction intersecting two SiC substrate peaks, namely, SiC(2–1.0) and the following (4–2.0) of the same family. For both orientations of the film, there are at least two reflection orders, i.e., {11.0} and {22.0} and {*n*0.0}, *n* = 2···4, which allow for a precise determination of the in-plane lattice parameter of the MBE-grown FGaT film to be *a* = 4.108[7] Å. This value is in excellent agreement with our RHEED value and also consistent with reported values for bulk crystals^22,41^ and MBE-grown films on mica.^42^ The origin of the bimodal distribution is not yet understood, but it may be related to different mechanisms occurring simultaneously during FGaT growth on graphene/SiC. These could include remote epitaxy, which is caused by the potential field of the polar SiC substrate, as well as direct vdW epitaxy of FGaT on graphene.^43^ Further investigations are required in order to confirm this hypothesis.

**Figure 2:**
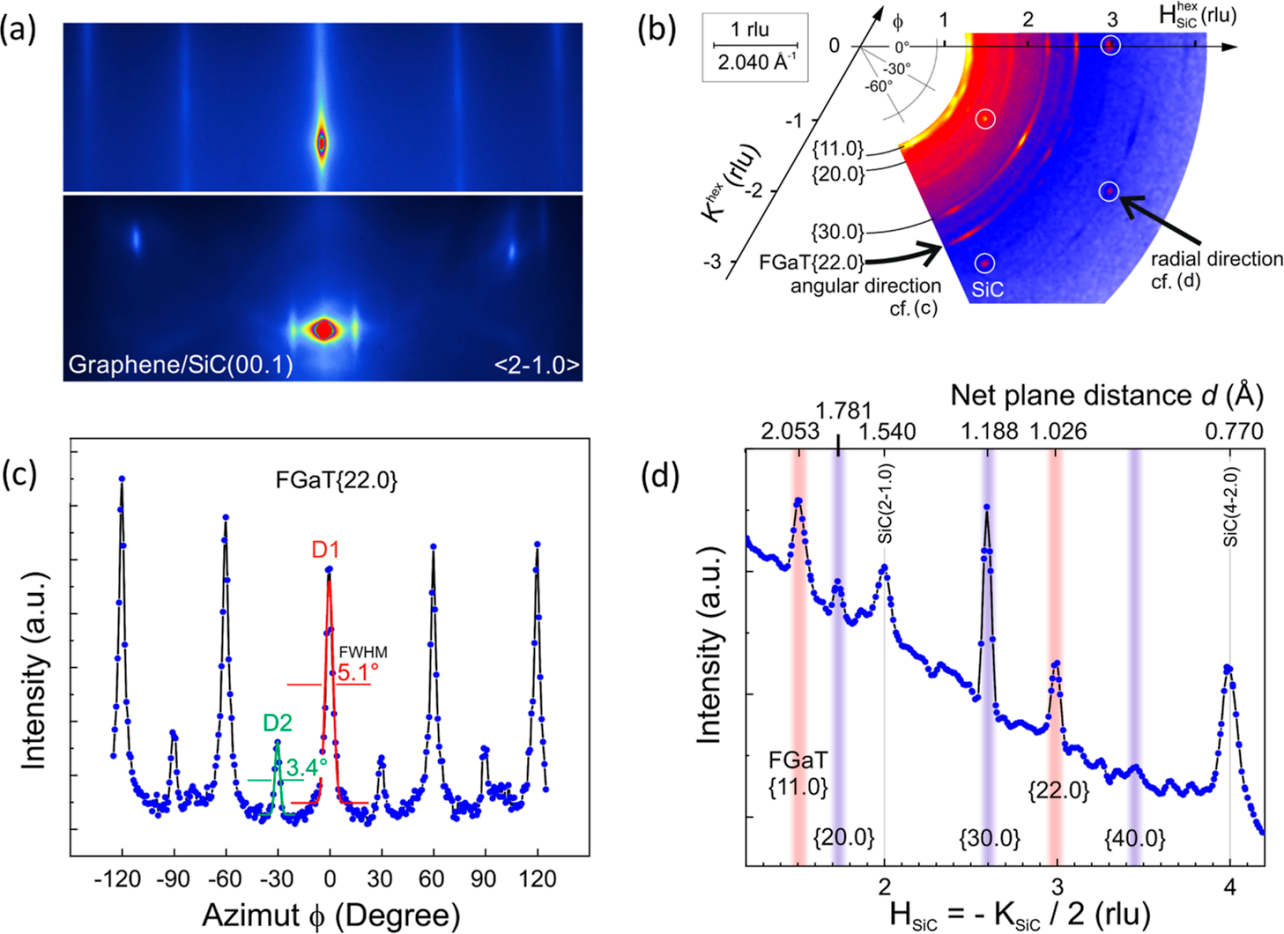
(a) In situ RHEED patterns obtained for the graphene/SiC template [bottom panel] and after the growth of a 10 nm thick FGaT film [upper panel]. Both patterns were taken perpendicular to the ⟨2–1.0⟩ direction of the SiC(00.1) substrate. (b) GID in-plane reciprocal space map of a 10 nm thick FGaT film on graphene/SiC(00.1). In addition to well-defined reflections from the SiC substrate (white circles), contributions from the FGaT film can be identified. (c) Azimuthal scan of the FGaT[22.0] peak along the angular direction. (d) The radial direction peaks corresponding to FGaT[11.0], [22.0], as well as FGaT[20.0], [30.0], [40.0] are denoted by red and purple vertical lines, respectively, as indicated in the in-plane reciprocal map (b).

Figure 3a shows an atomic force microscopy (AFM) image of a 10 nm thick FGaT film capped with a 7 nm thick Te layer. The line profiles displayed in the inset corresponding to markers 1 and 2 in Figure 3a represent the topography of the film along and perpendicular to the terrace step edges of graphene/SiC(00.1). A small value of root-mean-square (rms) roughness of 0.69 nm (along the line profile for marker 1) for this film indicates the growth of the FGaT film with very good uniformity without significant thickness variations or island formation. Also, no discernible discontinuities or pinholes in the film, e.g., close to step edges in graphene/SiC(00.1), could be observed. This is in contrast with our earlier reports on FGT grown on graphene/SiC(00.1),^44^ where such imperfections were more prevalent. Such observed morphological characteristics suggest a highly controlled growth process, resulting in FGaT films that homogeneously cover the underlying graphene/SiC template, including regions close to step edges where the transition between monolayer to bi­(few-)­layer graphene is normally present.^44^ This uniform film structure is crucial for maintaining consistent electronic and magnetic properties across the sample, which in turn is essential for further device processing and thus for applications.

**Figure 3:**
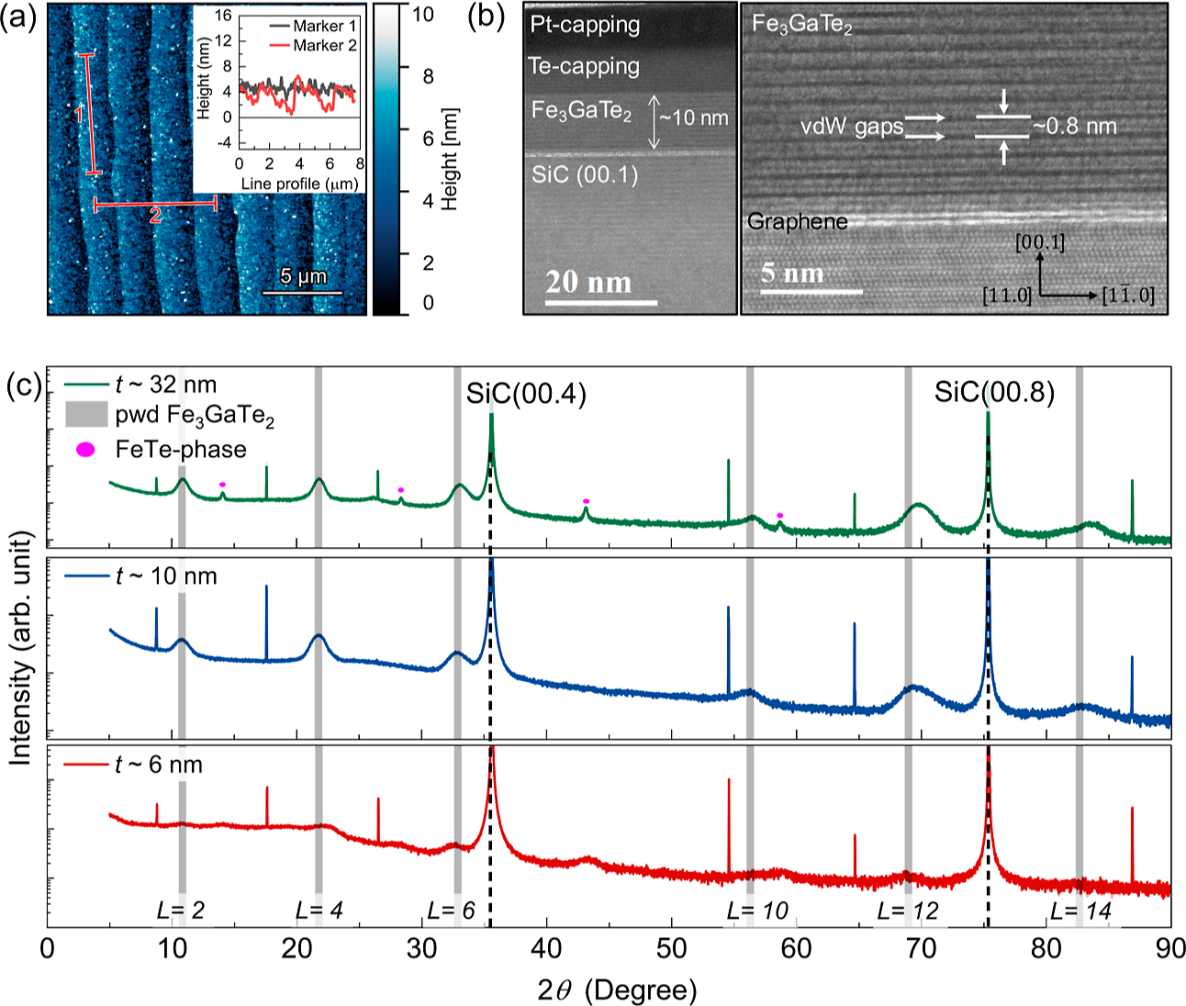
(a) AFM height image of a 10 nm thick FGaT film grown on graphene/SiC(00.1). The FGaT film is capped with a ∼7 nm thick Te layer. The inset shows the line profiles corresponding to markers 1 and 2. (b) Bright-field STEM images for the ∼10 nm thick FGaT film obtained along the [11.0] direction. While the left-side image shows the whole stacking, including the Pt/Te cappings, the right-side image shows a magnified region where it is possible to see the layered structure of FGaT and graphene. (c) The θ – 2θ scans for the 6, 10, and 32 nm thick FGaT films containing a series of reflections, whose positions are in good agreement with the calculated powder (pwd) pattern for FGaT (gray bars). The red dots indicate a small contribution from the tetragonal FeTe phase, which forms for the 32 nm thick FGaT case. The vertical dashed line represents the Bragg reflection for the SiC substrate.

The structure of the FGaT/graphene heterostructures was also assessed via cross-sectional scanning transmission electron microscopy (STEM). Figure 3b shows the bright-field images of a 10 nm thick FGaT film on graphene/SiC(00.1), which was capped with Pt/Te layers. The left-side image shows the smooth interface of the FGaT film to both the Te capping layer and the graphene/SiC(00.1) substrate, while the higher magnification image on the right side clearly reveals the uniform layered nature of the FGaT films (in that single layers are separated by vdW gaps) and its sharp interface to the underlying graphene. The thickness of each FGaT quintuple layer [formed by sequential Te/Fe/FeGa/Fe/Te slabs, see Figure 1a] is determined to be ∼0.8 nm, which is in agreement with the values obtained for FGaT bulk crystals.^22^

XRD θ – 2θ scans were performed to probe the out-of-plane structure of the FGaT films [Figure 3c] grown on the graphene/SiC(00.1) substrate. The presence of (00.*L*) reflections (where *L* = 2, 4, ..., 14) for 6 and 10 nm thick FGaT indicates the formation of a pure crystalline FGaT phase. However, the presence of additional small reflections (marked by magenta dots) for the case of the 32 nm thick film revealed the formation of tetragonal FeTe in addition to the FGaT phase, with its *c*-axis aligned parallel to the SiC [00.1] direction. Similar to what has been previously observed for MBE-grown Fe_5–*x*_GeTe_2_ films,^19^ we suggest that the small islands located on the surface of the 32 nm FGaT film (see Figure S2) are composed of the tetragonal FeTe phase. The reason for their formation is not well understood and needs further investigation. The Bragg peak positions and relative intensities of the FGaT reflections closely matched the numerically calculated powder diffraction pattern (indicated by gray bars), corroborating the structural integrity of the FGaT films. The out-of-plane lattice parameters were determined using the Debye–Scherrer formula,^45^ and they were found to be around 16.39 Å, 16.41 Å, and 16.30 Å for the 6, 10, and 32 nm thick FGaT films, respectively, which is in excellent agreement with our STEM results [note that a single FGaT layer corresponds to 1/2 of the unit cell; see Figure 1a]. These values are also consistent with previously reported results for both FGaT bulk crystals^20^ and FGaT thin films.^42^ It is noteworthy that these XRD measurements were performed on Te-capped FGaT films, which may have implications for the observed lattice parameters and peak intensities. The presence of the Te capping layer could potentially influence the strain state of the underlying FGaT film, particularly in the case of ultrathin samples.

### Magnetic Properties

Single scanning nitrogen vacancy (NV) center-based microscopy was used to probe the magnetic domain structure of the MBE-grown FGaT films. Figure 4a shows an NV field map collected for the 32 nm thick FGaT film. This magnetic field map was measured at 300 K, indicating the formation of a stable magnetic domain structure at room temperature. Static magnetization measurements were performed using a SQUID (superconducting quantum interference device) magnetometer to investigate the magnetic properties of FGaT films, namely, coercive field (*H*_C_), Curie temperature (*T*_C_), and saturation magnetization (*M*_S_). In Figure 4b, the magnetization hysteresis loop for a 10 nm thick FGaT film was measured at different temperatures by sweeping the magnetic field in out-of-plane (*H*∥*c*) ranging between +2 and −2 T. A strong linear diamagnetic contribution signal originating from the graphene/SiC(00.1) template was subtracted from the curves. Besides a large coercivity up to room temperature, a small magnetic contribution also adds and leads to a two-step switching feature in the hysteresis loop. This two-step switching behavior in magnetic thin films can arise from multiple reasons, such as (a) partial surface oxidation of the film, (b) magnetic impurities from the bare substrate, (c) intercalation of Fe atoms into the vdW gap, and (d) the presence of multiple magnetic domains with different coercivities. We ruled out the possible origin of the two-step feature from oxidation of the film (protected with the Pt/Te capping layer) and the contribution of the graphene/SiC substrate (as explained in Supporting Information S3). Therefore, we suggest that this feature with negligible *H*_C_ could be related to the existence of multiple magnetic domains^42,46^ or intercalation effects.^46–49^ Further investigations are needed to clarify this aspect in more detail. In Figure 4c, the extracted values of *H*_C_ and *M*_S_ are plotted as a function of temperature. The large values of *H*_C_ and *M*_S_, in particular at 10 K, indicate a strong PMA for the MBE-grown FGaT, which is also confirmed by hysteresis loops obtained via magneto-transport and X-ray magnetic circular dichroism (XMCD) measurements (see discussions later).

**Figure 4:**
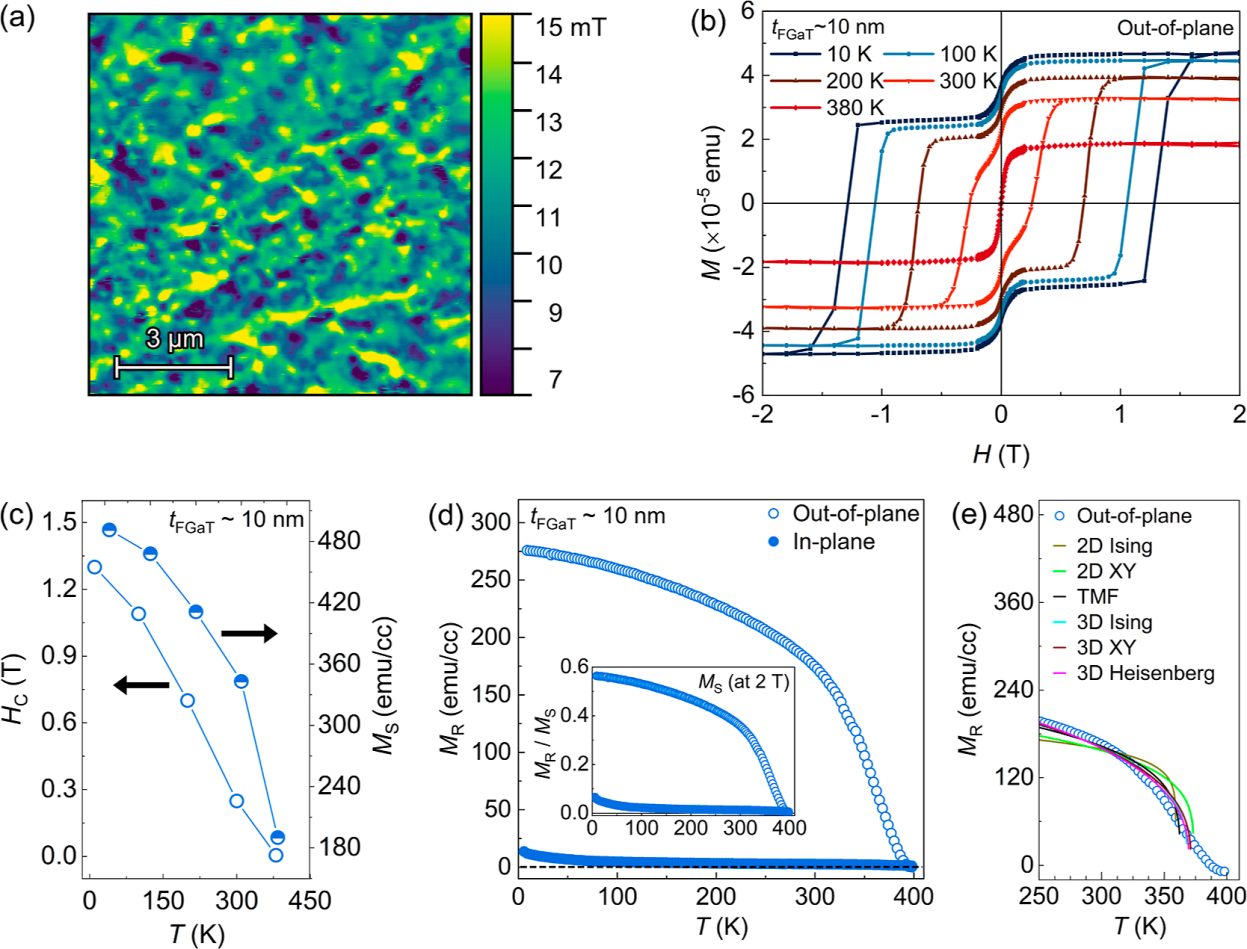
(a) NV field map measured at room temperature for a 32 nm thick FGaT film on graphene/SiC(00.1) using scanning–NV center microscopy. (b) Magnetization (*M*) versus magnetic field (*H*) hysteresis loop measured at different temperatures for a 10 nm thick FGaT film on graphene/SiC(00.1). (c) Extracted values of *H*_C_ and *M*_S_ as a function of temperature. (d) Remanent magnetization, *M*_R_, as a function of temperature for both in-plane (*H*∥*ab*) and out-of-plane (*H*∥*c*) configurations; the insets represent the corresponding *M*_R_/*M*_S_. (e) *M*_R_ as a function of temperature for the out-of-plane (*H*∥*c*) configuration plotted together with fittings obtained with eq 1 using different critical exponents.

SQUID also allowed us to study the temperature dependence of remanence magnetization (*M*_R_). For these measurements, we first applied a large positive field of +5 T to magnetize the FGaT film completely. After the field was set to 0 T in linear mode, the *M*_R_ was measured within the temperature range of 5–400 K. Figure 4d shows the results obtained for both in-plane (*H*∥*ab*) and out-of-plane (*H*∥*c*) field configurations. For the out-of-plane case, it is observed that the *M*_R_ value reduces only ≈35% (from 276 emu/cc to 180 emu/cc) from 5 to 300 K, which indicates a good thermal stability of the out-of-plane magnetization. The inset plot shows the normalized *M*_R_/*M*_S_, and the *M*_S_ value used here is taken for a 2 T field. Considering the extrapolation of the out-of-plane (*M*_R_) to zero, a high *T*_C_ value around 390 K can be deduced. In addition, the *T*_C_ value was also estimated by fitting the out-of-plane *M*_R_–*T* curve shown in Figure 4e using the equation below:^50^

1 $$M_{R}=M_{0}{\left(1-\frac{T}{T_{C}}\right)}^{\beta }$$

where β is the magnetic critical exponent for which we considered different models, namely, the 3D Heisenberg, 3D *XY*, 3D Ising, tricritical mean field (TMF), 2D *XY*, and 2D Ising. For the 10 nm FGaT film, the extracted values of *T*_C_ and β are 377 ± 0.7 K and 0.38, respectively. For β = 0.38 [found for 10 nm thick FGaT, which is approximately 6 unit cells thick, see Figure 1d], the curve shows a close fit to the 3D Heisenberg model, which suggests 3D ferromagnetism. For the 6 nm FGaT film (∼4 unit cells), we also measured the out-of-plane *M*_R_–*T* curve and fitted it with eq 1 (see Figure S4). The extracted value of β = 0.21 in this case indicates a transition to 2D ferromagnetism.^49,51,52^ Both films show a “tail-like” behavior of the *M*_R_ value near the transition temperature, which is more pronounced for the 6 nm thick case. This is consistent with previous reports on 2D ferromagnets such as FGT flakes^49,53^ and MBE-grown FGaT films^42^ having similar thicknesses. The origin of the tail-like behavior of the magnetization curve can be explained by positive-feedback mean-field modification of classical Brillouin magnetization theory.^54^ In any case, the *T*_C_ obtained from the SQUID investigations confirm that ferromagnetic order persists well above 350 K in our MBE-grown films, which is comparable or higher to what has been shown for bulk single crystals (and flakes),^20,29^ as well as MBE films grown on mica.^42^

### Anomalous Hall Effect

Temperature-dependent magneto-transport measurements were performed for FGaT/graphene heterostructures with different FGaT thicknesses (*t*_FGaT_ = 6, 10, and 32 nm), as shown in Figure 5a. In ferromagnetic materials, the transverse Hall resistance (*R*_*xy*_), under the application of a perpendicular magnetic field (*H*), is given by eq 2 ^55,56^

2 $$R_{xy}=R_{OHE}+R_{AHE}$$

where the first and second terms represent the contribution from the ordinary Hall effect (OHE, *R*_OHE_ = *R*_0_ *H*) and the anomalous Hall effect (AHE, *R*_AHE_ = μ_0_ *R*_S_ *M*_Z_), respectively. *R*_0_ and *R*_S_ are the coefficients of ordinary and anomalous Hall resistance, respectively; μ_0_ is the vacuum permeability and *M*_Z_ is the out-of-plane magnetization. Figure 5b displays *R*_*xy*_ measured at 10 K for samples having *t*_FGaT_ = 6, 10, and 32 nm. For all cases, the AHE is detected as a square-shaped hysteresis loop. In contrast to the double loop *M*–*H* feature obtained from SQUID, we observed a single loop shape AHE hysteresis for all the thicknesses, which confirms the stoichiometric FGaT with a robust PMA.^42^ We attribute the absence of a double-loop behavior in the AHE hysteresis curves to the nonhomogeneous nature of potential minor secondary phases or magnetic domains, for example, involving localized Fe intercalation in the vdW gaps, which can only be detected by SQUID. Such discrepancy between SQUID and magneto-transport data is consistent with observations from other groups for Fe_3_GaTe_2_ ^42^ and Fe_3_GeTe_2_.^46,48^ Furthermore, the AHE is superimposed on an OHE contribution that has a linear field dependence due to the metallic nature of both the FGaT and the underlying graphene^19,57^ (see the discussions later). Furthermore, the coercive fields (*H*_C_) are very similar for all samples, while a larger value of *R*_AHE_ is measured for the thinnest FGaT film. This expected behavior of exhibiting more pronounced *R*_AHE_ with lower thickness has previously been observed, not only for FGaT^29,42^ but also for FGT.^58^

**Figure 5:**
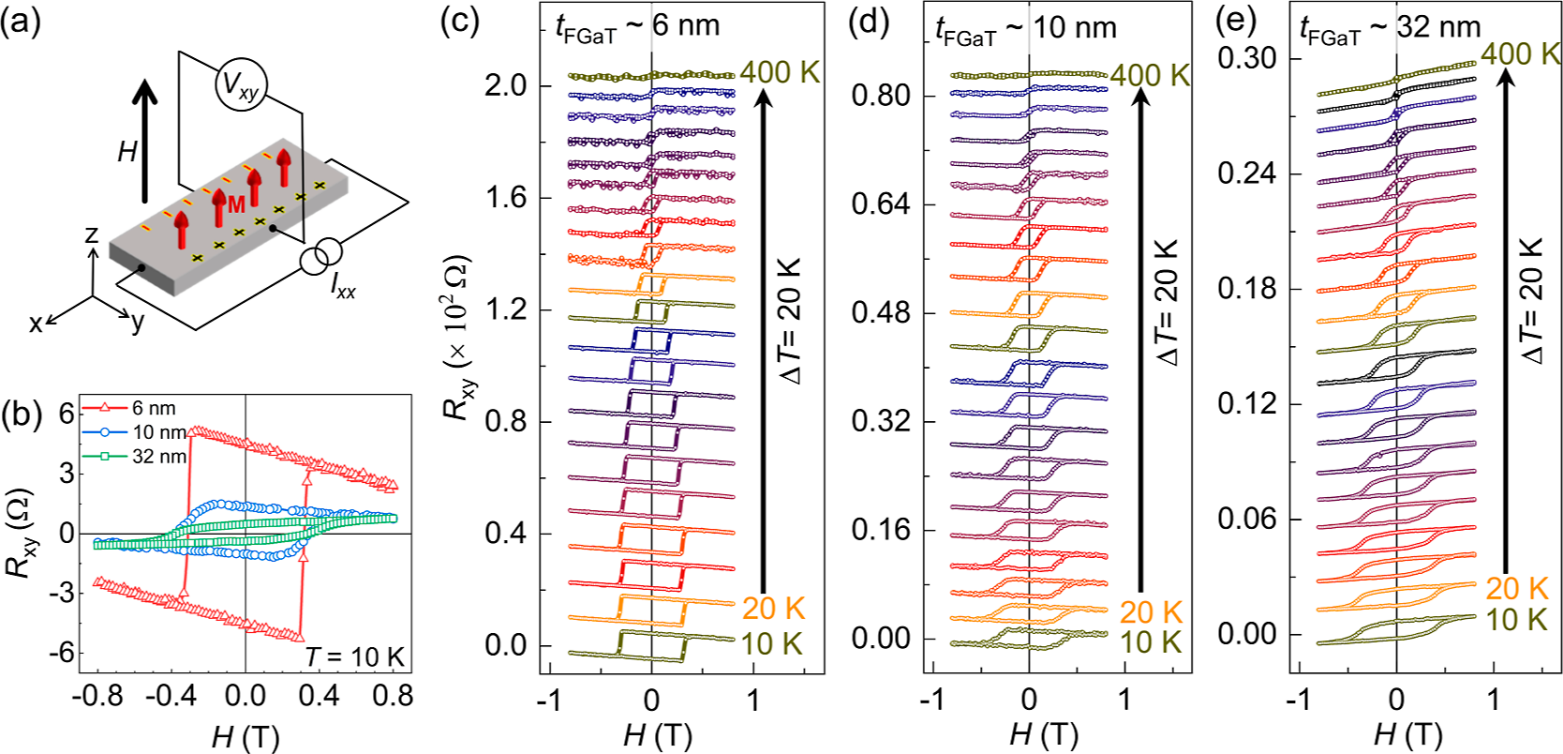
(a) Schematic of the magneto-transport measurement where the Hall voltage (*V*_*xy*_) is measured along the *y*-direction, while the direct current (*I*_*xx*_) flows in the *x*-direction, and the external magnetic field (*H*) is swept along the *z*-direction. (b) Transverse resistance (*R*_*xy*_) versus external magnetic field (*H*) hysteresis loops for 6 nm, 10 nm, and 32 nm thick FGaT films on graphene/SiC(00.1) measured at 10 K. The temperature-dependent *R*_*xy*_ was measured from 10 to 400 K at steps of 20 K for (c) 6 nm, (d) 10 nm, and (e) 32 nm thick FGaT films on graphene/SiC(00.1).

The temperature dependence of *R*_*xy*_(*H*) is shown in Figure 5c–e for the FGaT samples with three different thicknesses. One can see that the AHE vanishes only at 400 K. This confirms the existence of ferromagnetic order with PMA at and above room temperature, consistent with the NV-center and SQUID results. The temperature dependence of *R*_AHE_ and *H*_C_ is illustrated in Figure 6a,b, respectively. Considering the extrapolation of both *R*_AHE_ and *H*_C_ quantities to zero, we tentatively conclude that the transition temperature *T*_C_ of the FGaT films is around 400 K. The value of *T*_C_ obtained for our films is larger than what has recently been reported for FGaT MBE films grown on GaAs,^59^ but similar to FGaT films (grown on mica)^42^ and exfoliated flakes^20^ of comparable thicknesses.

**Figure 6:**
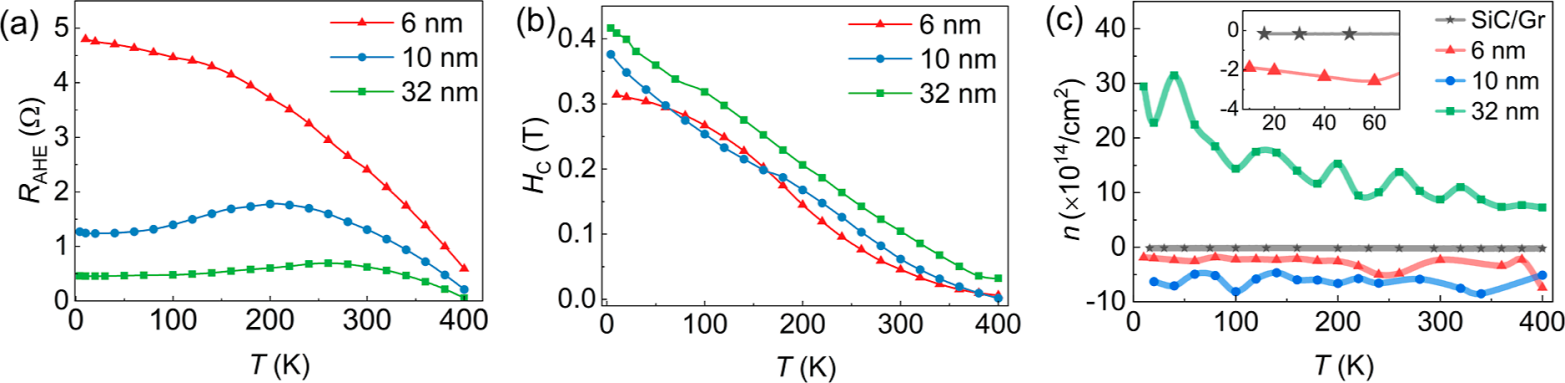
(a) Anomalous Hall resistance *R*_AHE_ as a function of temperature for FGaT films with different thicknesses. (b) The dependence of the extracted value of the coercive field *H*_C_ from the transverse resistance contribution as a function of temperature. The solid lines are guides to the eye. (c) Dependence of the hole/electron carrier density on the temperature for FGaT films with different thicknesses and also the pristine graphene/SiC(00.1) template denoted by a star symbol. The average value of electron/hole carrier density (*n*_e/h_) was determined from the plot; the inset in (c) represents the zoom value for the graphene/SiC template compared to the 6 nm thick FGaT film.

Finally, using the relation $R_{0}=\frac{1}{n_{e/h}\left|q\right|}$ , the charge carrier type [*q*: electron/hole (e/h)] and carrier density (*n*_e/h_) were determined for the three different thicknesses. Figure 6c shows the extracted value of *n*_e/h_ as a function of temperature from 400 K down to 10 K. For the 32 nm thick FGaT film, we observed an increase in the p-type carrier density with the decrease in temperature. It is believed that the electronic band structure of FGaT is similar to FGT, only by considering the FGaT structure doped with 1 hole per formula unit.^22,49^ Therefore, our 32 nm thick FGaT film shows hole-type conductive behavior over the whole temperature range of 10–400 K, which is consistent with previous reports.^20^ In contrast, for the heterostructures containing 6 and 10 nm FGaT films, an n-type transport behavior was measured over the whole temperature range. We attribute this change in the carrier type in our thinner films to a larger contribution of the underlying graphene layer to the electronic transport. Graphene on SiC(00.1) is known to be intrinsically n-type doped^60,61^ and to act as a high-mobility transport channel when combined into vdW heterostructures with 2D ferromagnetic metals such as Fe_3_GeTe_2_ and Fe_5–*x*_GeTe_2_.^19,44^ Thus, while the transport behavior in the thickest FGaT/graphene sample is dominated by FGaT, an enhanced contribution from graphene with a two-transport channel occurs in the samples with reduced FGaT thickness. In this case, the value and sign of the slope associated with the OHE will depend on the relative conductivities of both FGaT and graphene and will not correspond to the value of a single material. Furthermore, the secondary tetragonal FeTe phase identified by XRD in the 32 nm FGaT film is expected to have negligible contribution to the electronic properties, due to the absence of transverse resistance^62^ and the isolated island morphology of FeTe rather than a continuous layer (see Figure S2), similar to what has been previously reported for Fe_5–*x*_GeTe_2_ films.^19^ The extracted values of *n*_e/h_ measured at 10 and 300 K for three different thicknesses of the FGaT/graphene heterostructure are summarized in Table 1 together with the values for *R*_AHE_ and *H*_C_. Note that the value of *n*_e/h_ measured for a bare graphene/SiC(00.1) template is mentioned as a footnote of Table 1 for reference.

**Table 1:** Value of *R*_AHE_, *H*_C_, and *n*_e/h_ Measured at Temperatures of 10 and 300 K for Three Different Thicknesses of FGaT and the Graphene/SiC Template^a^

sample thickness	*R*_AHE_ (Ω)		*H*_C_ (T)		*n*_e/h_ (×10^14^/cm^2^)	
	10 K	300 K	10 K	300 K	10 K	300 K
FGaT (6 nm)	4.75	2.40	0.31	0.05	–2.03	–2.32
FGaT (10 nm)	1.24	1.31	0.34	0.06	–6.29	–6.51
FGaT (32 nm)	0.45	0.62	0.41	0.10	+29.4	+8.71

a The value of *n*_e/h_ for the graphene/SiC template is found to be −0.19 × 10^14^/cm^2^.

### X-ray Absorption Spectra and X-ray Magnetic Circular Dichroism

The magnetic properties of FGaT thin films were also studied using XMCD. The measurements were performed on 6 and 10 nm thick FGaT films at normal incidence (NI, θ = 90°) at the Fe *L*_2,3_ absorption edges.

Figure 7a,b shows the XMCD hysteresis loops measured in NI mode at two different temperatures, 15 and 300 K. These XMCD loops were taken at a photon energy of 706.7 eV (corresponding to the *L*_3_ absorption edge of Fe^2+^), along with a pre-edge taken at 698.0 eV for background. At 15 K (300 K), the values of *H*_C_ for the 6 and 10 nm FGaT films were found to be ∼2 T (0.2 T) and ∼1.15 T (0.25 T), respectively. These values are in good agreement with the results obtained via SQUID. The large *H*_C_ values for both films, in particular, at 15 K, confirm the strong PMA in our MBE-grown FGaT layers. In Figure 7c,d, the XMCD spectra (μ_m_) were extracted by taking the differences of the X-ray absorption spectra (XAS) spectra (μ_+_ and μ_–_) measured at 15 K with a constant magnetic field (±*H* = 2.5 T) for both thicknesses. The XAS and XMCD intensities are denoted as μ_+_, μ_–_, and μ_m_ on the left and right axes of the plot, respectively. Similarly, for both samples, the XMCD spectra were obtained over the temperature range from 15 to 400 K. By applying the sum rule analysis,^63–65^ the values of effective spin magnetic moment (*μ*_S_^eff^), orbital magnetic moment (μ_l_), total magnetic moment (μ_total_) were determined (see Supporting Information S5). In Figure 7e, the extracted values of μ_total_ for both film thicknesses are plotted as a function of temperature. At 15 K, the values of *μ*_S_^eff^, μ_l_, and μ_total_ for the 10 nm [6 nm] thick FGaT film were found to be 1.53 μ_B_/Fe [0.74 μ_B_/Fe], 0.14 μ_B_/Fe [0.04 μ_B_/Fe], and 1.67 μ_B_/Fe [0.78 μ_B_/Fe], respectively. At 300 K, these values decreased to 1.13 μ_B_/Fe [0.45 μ_B_/Fe], 0.03 μ_B_/Fe [0.02 μ_B_/Fe], and 1.16 μ_B_/Fe [0.47 μ_B_/Fe], respectively. Notably, the value of μ_total_ at 300 K for the 10 nm film (1.16 μ_B_/Fe) is in excellent agreement with values previously reported for FGaT bulk crystals using SQUID magnetometry.^20^ Since the μ_total_(*T*) curves were recorded under a saturating magnetic field of ±2.5 T, the temperature-dependent XMCD curves show a qualitative agreement with the *M*_S_(*T*) curve obtained from SQUID [see Figure 4c], which was also extracted from the saturation field regime (2 T). The ratio of orbital to spin magnetic moment (μ_l_/*μ*_S_^eff^) was determined to be 0.092 [0.058] at 15 K and 0.027 [0.049] at 300 K for the 10 nm [6 nm] thick FGaT films. The nonzero μ_total_ for both thicknesses up to 400 K, as shown in Figure 7e, reveals a stable ferromagnetic ordering of Fe atoms in the FGaT layer sustaining a robust PMA above room temperature. This temperature-dependent behavior of μ_total_ for both FGaT thicknesses further confirms the high *T*_C_ ∼ 400 K, consistent with the results obtained from *M*–*T* curves by SQUID and *R*_AHE_–*T* curves obtained via magneto-transport. Importantly, we emphasize that XMCD not only probes the near-surface region of the film but is also element-specific, being measured at a fixed photon energy at the Fe *L*_2_-edge. In contrast, SQUID captures the net magnetic response from all magnetic contributions across the entire film stack, including substrate magnetic impurities. Despite having these differences in SQUID, Hall, and XMCD curves, we can still make a qualitative comparison to demonstrate the transition temperature in these 2D ferromagnetic films. Therefore, these results on high-quality vdW-based heterostructures are pivotal for advancing next-generation nonvolatile memory technologies, including magnetic random-access memory and skyrmion-based data storage.^28,66^

**Figure 7:**
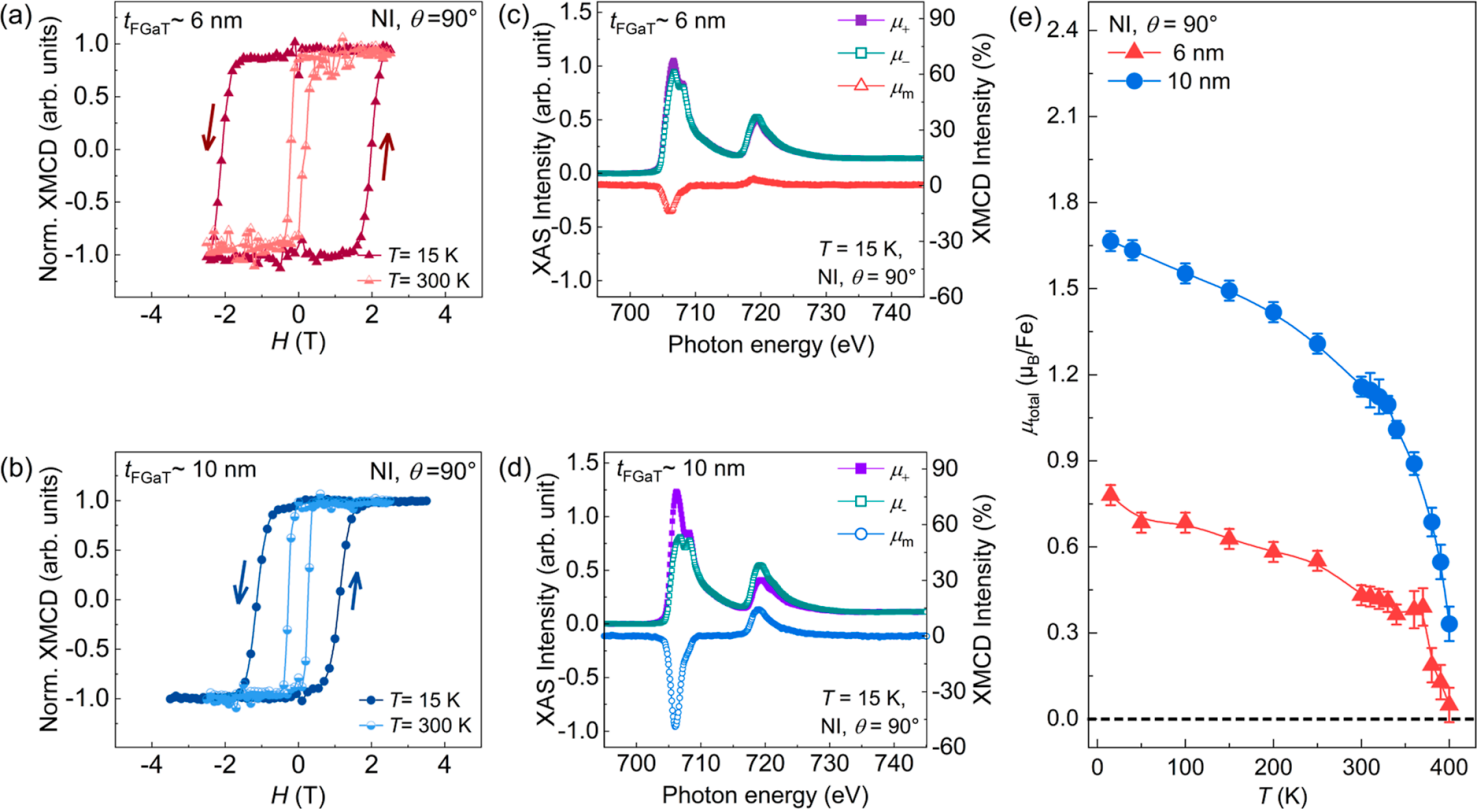
XMCD hysteresis loops probed at the Fe *L*_3_ edge were measured at temperatures of 15 and 300 K in NI mode (θ = 90°, i.e., the external magnetic field is swept along the *c*-axis of the FGaT layer) for a (a) 6 nm thick FGaT film and (b) 10 nm thick FGaT film. The XAS and XMCD plots measured at 15 K in NI mode for a (c) 6 nm thick FGaT film and (d) 10 nm thick FGaT film. (e) The closed triangle and circle symbols represent the total magnetic moment (μ_total_) plotted as a function of temperature for 6 and 10 nm thick FGaT films, respectively, measured in NI geometry.

## Conclusion

We have demonstrated the high-quality, large-scale epitaxial growth of 2D ferromagnetic Fe_3_GaTe_2_ films directly on graphene/SiC(00.1) substrates. Structural characterization confirms the excellent crystalline quality, homogeneous surface, and sharp interface of the FGaT/graphene vdW heterostructure. Magnetic and transport measurements reveal robust PMA with a high Curie temperature reaching 400 K. These findings are further substantiated by XMCD measurements, where perpendicular magnetic moments were probed under NI geometry. Additionally, the spin, orbital, and total magnetic moment contributions were extracted from X-ray absorption spectroscopy performed over a wide temperature range (10 to 400 K). At 300 K, we obtained the μ_total_ to be 1.16 μ_B_/Fe for our 10 nm thick FGaT film, which is consistent with the previous reports on FGaT bulk crystals. Our findings underscore the potential of FGaT as a platform for high-performance spintronic devices based on 2D heterostructures, with potential applications in future data storage, processing technologies, and quantum computing.

## Experimental Method

### Sample Preparation

We grew epitaxial graphene on semi-insulating 4H-SiC(00.1) substrates using the SiC surface graphitization method.^40^ Before this, the substrates were chemically cleaned under ultrasonication using *n*-butyl acetate, acetone, and isopropanol and later dried with N_2_ gas. This was followed by annealing in a forming gas environment consisting of 95% Ar and 5% H_2_ gases at 1400 °C for 15 min to obtain smooth, well-defined stepped surfaces. Then, the synthesis of smooth layers of epitaxial graphene was realized in an atmosphere of Ar gas at a temperature of 1600 °C for 15 min, similar to our previous work.^19,44^

The high-quality large-area epitaxial Fe_3_GaTe_2_ thin films were successfully grown on graphene/SiC(00.1) templates, in ultrahigh vacuum (UHV) conditions using MBE. Before the film growth, the templates were degassed at 450 °C for 1 h in the UHV chamber to remove surface contaminants. High-purity elemental Fe, Ga, and Te were evaporated from separate effusion cells. Their temperatures were chosen to realize films with the desired composition of Fe_3_GaTe_2_, and the fluxes for each element were obtained by measuring the beam equivalent pressure employing a pressure gauge. The growth chamber was maintained at a base pressure below 1 × 10^–10^ mbar and a working pressure below 1 × 10^–9^ mbar. An optimized substrate temperature of 370 °C was chosen to get a high-quality crystalline phase of the FGaT film. After reaching the desired thickness, ranging from 6 to 32 nm, the samples were allowed to cool to room temperature (RT).

Later, at RT, a 5–7 nm Te capping layer was deposited in order to minimize the oxidation of the FGaT films upon air exposure. For some of the samples, an additional Pt capping layer was deposited in situ, also at RT, on top of the Te capping layer to avoid oxidation of the Te layer. The entire growth process was monitored by using in situ RHEED to assess the crystalline quality of the growing films. A low growth rate of 0.17 nm/min was employed during FGaT growth.

### Composition and Structural Characterization

The accurate value of the chemical composition of the FGaT films was determined with the help of the RBS technique using 1.7 MeV He^+^ ions with a scattering angle of 170°. From the RBS measurements, the chemical composition of Fe:Ga:Te was found to be around 3.15:1:1.47, thus revealing a small Fe excess and Te deficiency in comparison to stoichiometric FGaT. The surface morphology and the film thickness were examined by atomic force microscopy (AFM), conducted in the standard tapping mode. For the STEM measurements, the lamellae were prepared using the Thermo Fisher Helios 5 UX focused ion beam (FIB) system, which is equipped with a Ga^+^ ion beam source operating in the energy range from 1 to 30 keV. Using a nanomanipulator, the cross-sectional lamella was lifted out according to standard procedures and transferred to a grid. The lamella was then thinned to the desired shape and thickness and, in a final step, polished with a low-energy ion beam to minimize the surface damage caused during FIB preparation. The lamellae were then immediately inserted into the TEM for structural observation to minimize oxidation of the iron-containing layer. The STEM measurements were carried out in a JEOL 2100 F field emission electron microscope at an acceleration voltage of 200 kV. The individual thickness of each layer in the film stack was further characterized by X-ray reflectivity (XRR). Both XRR and out-of-plane XRD (θ – 2θ scan) were performed using a PANalytical X’Pert Pro MRD diffractometer with Cu Kα radiation (λ = 1.5418 Å). To investigate the in-plane crystallographic properties, grazing incidence diffraction (GIXRD) was performed at synchrotron beamline BM25B of the European Synchrotron Radiation Facility (ESRF) in Grenoble. A monochromated X-ray beam was used with a photon energy of 18 keV. A grazing incidence angle close to that of total external reflection makes this technique extremely surface sensitive.

### Magnetization Measurement

The static magnetization measurements were performed using a Quantum Design MPMS3 SQUID magnetometer at different temperatures between 10 and 400 K by sweeping the applied magnetic field up to 2 T. Magnetization vs temperature measurements were performed in both in-plane and out-of-plane configurations for the same sample pieces. Magnetic field sweeps were performed in a persistent mode; the temperature sweeps were performed at a rate of 5 K per minute.

The stray field image of the magnetic domain was acquired on a commercially available single nitrogen-vacancy (NV) scanning microscope (QZabre—QST).

### Anomalous Hall Measurement

Anomalous Hall measurements were conducted on a rectangular strip [0.5 × 1 cm] piece of Fe_3_GaTe_2_ on graphene/SiC(000), which was bonded to a chip carrier using aluminum contact wires. A constant current of 100 mA was applied along the longest side of the piece in the *x*-direction. The transverse voltage (*V*_*xy*_) was recorded perpendicular to the direct current (*I*_*xx*_) flow using two centrally positioned contacts on the sample strip along the *y*-axis. Experiments were conducted across a wide temperature range, spanning from 10 to 400 K, in a high-vacuum environment with pressure maintained around 10^–6^ mbar. The external magnetic field was swept between ±0.8 T perpendicularly to the sample surface (along the *c*-axis).

### XAS and XMCD Measurements

The XAS measurements were conducted at the VEKMAG end-station on the PM2 beamline at BESSY II. This facility provides a vector magnetic field of up to 9 T along the beam direction, 2 T in the horizontal plane, and 1 T in all directions, within a temperature range of 2–500 K. The spectra were recorded using the total electron yield (TEY) method, which measures the drain current as a function of X-ray photon energy. TEY, being surface-sensitive with an electron escape depth of ∼3 nm, selectively provides information on surface magnetic properties.^67^ A magnetic field of up to ±2.5 T was applied along the beam direction to magnetically polarize the sample during the XMCD experiments. The XMCD signal was obtained by calculating the difference between the XAS signals measured under two opposite magnetic fields.^68^ The sum rule analyses were performed on the integral signal of XAS and XMCD spectra to calculate the contribution from spin and orbital magnetic moment (see Supporting Information S5).

## Supplementary Material



## Notes

Section on composition analysis with full RBS spectrum for all the elements contained in Pt/Fe_3_GaTe_2_ (10 nm)/graphene/SiC heterostructures, AFM topography for the 32 nm thick Fe_3_GaTe_2_ film with FeTe impurity, SQUID loops with the bare template, i.e., graphene/SiC, temperature-dependence of remanent magnetization/saturation magnetization (*M*_R_/*M*_S_) for the 6 nm Fe_3_GaTe_2_ film, and XAS and XMCD sum rule analysis (PDF)
