# Supplementary material for: Above-Room-Temperature Ferromagnetism in Large-Scale Epitaxial Fe3GaTe2/Graphene van der Waals Heterostructures
Source: ACS Nano. 2025 Oct 21;19(43):37561–71. doi: 10.1021/acsnano.5c07732 (PMC12593369; doi:10.1021/acsnano.5c07732)
Supplement: Supplementary file 1 [file nn5c07732_si_001.pdf]

Supporting Information for: Above-Room-Temperature Ferromagnetism in Large-scale Epitaxial Fe_3GaTe_2/graphene Van der Waals Heterostructures

Tauqir Shinwari,[*,†,‖] Kacho Imtiyaz Ali Khan,[*,†,‖] Hua Lv,[†] Atekelte Abebe Kassa,[†] Frans Munnik,[‡] Simon Josephy,[¶] Achim Trampert,[†] Victor Ukleev,[§] Chen Luo,[§] Florin Radu,[§] Jens Herfort,[†] Michael Hanke,[†] and Joao Marcelo Jordao Lopes[*,†]

†*Paul-Drude-Institut für Festkörperelektronik Leibniz-Institut im Forschungsverbund Berlin e.V 10117 Berlin, Germany.*

‡*Helmholtz-Zentrum Dresden-Rossendorf, Institute of Ion Beam Physics and Materials Research, Bautzner Landstrasse 400, 01328 Dresden, Germany.*

¶*QZabre AG, Neunbrunnenstrasse 50, 8050 Zürich, Switzerland.*

§*Helmholtz Zentrum Berlin for Materialien und Energie, Albert-Einstein Straße 15, 12489 Berlin, Germany.*

‖*These authors contributed equally to this work*

E-mail: shinwari@pdi-berlin.de; khan@pdi-berlin.de; lopes@pdi-berlin.de

S1: Composition analysis: Rutherford backscattering (RBS)

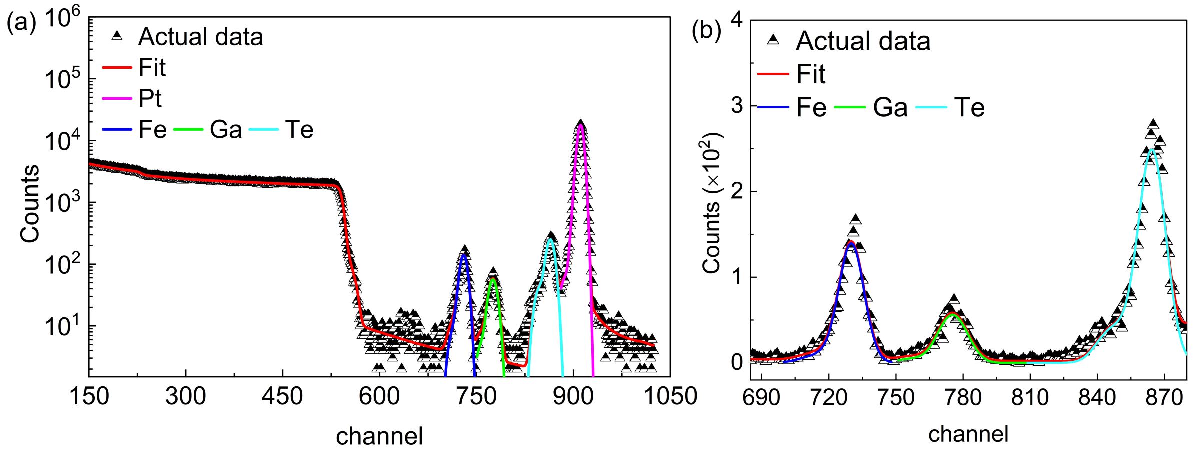

Figure S1: (a) Full RBS spectrum for all the elements contained in Pt/FGaT(10 nm)/graphene/SiC heterostructures and (b) the zoomed image corresponds to the region for the elements of Fe, Ga, and Te. The symbols and lines represent the experimental values and fit to the spectrum, respectively.

S2: Thick 32 nm FGaT film with FeTe impurity.

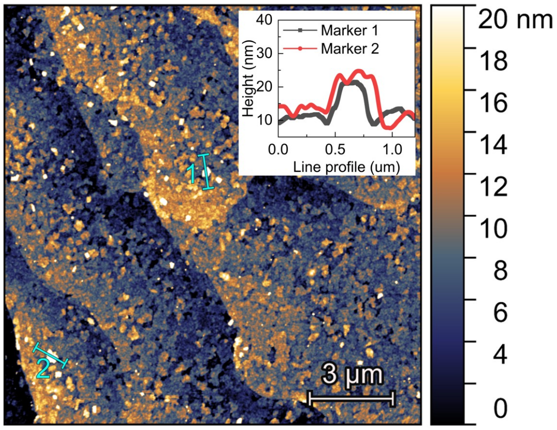

Figure S2: AFM topography image for the 32 nm thick FGaT film. The inset plot shows the height profiles obtained for the markers 1 and 2 shown in the main image.

Fig. S2 shows an atomic force microscopy (AFM) image obtained for the 32 nm FGaT film, which is different from the thinner films (see Fig. 3a, main text). It is possible to observe the existence of small islands all over the surface. The inset plot shows the height profiles corresponding to the markers 1 and 2 in the image, revealing that the islands can be as high as about 10 nm, similar to the case of $Fe_{5-x}GeTe_2$ films grown on graphene/SiC. [1] We suggest these islands are composed of tetragonal FeTe. Note that tetragonal FeTe is an antiferromagnetic below 70 K (Néel temperature), and it becomes paramagnetic above 70 K[2]. Therefore, its contribution to the overall transverse resistance is expected to be negligible. Moreover, the temperature dependence and field response of our transport data are consistent with those reported for pure FGaT, with no signatures of FeTe-related anomalies. Hence, we conclude that the secondary FeTe phase observed for the 32 nm thick FGaT film does not contribute to the magneto-transport properties of the FGaT/graphene heterostructures.

S3: SQUID loops with bare graphene/SiC

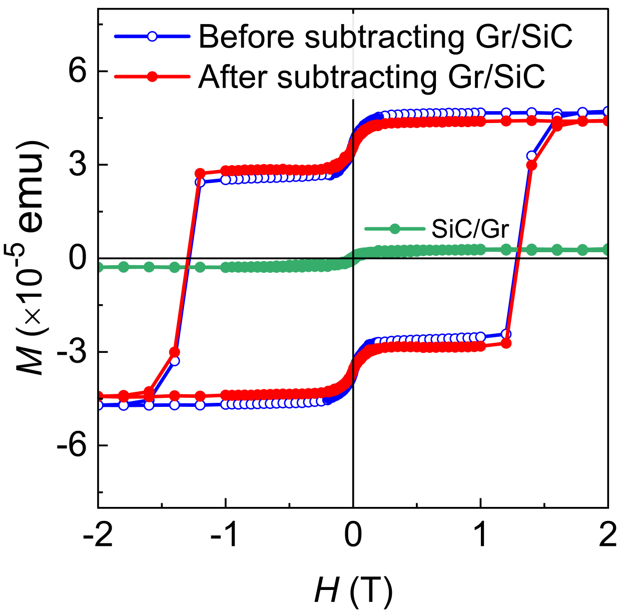

Figure S3: Magnetic moment (M) versus magnetic field (H) hysteresis loop measured at 2 K, in out-of-plane geometry for FGaT(10 nm)/Gr/SiC before (blue symbol) and after (red) subtracting contribution from Gr/SiC template (green).

To rule out the possibility of substrate contributions to the net magnetic moment, we performed SQUID measurements on the bare graphene/SiC (Gr/SiC) substrate. Fig. S3 shows the MH loops for both FGaT/Gr/SiC(00.1) (blue symbols) and the bare Gr/SiC(00.1) template (green symbols) measured at 2 K. We observed that after subtracting the small magnetic contribution of the substrate (red symbols), the two-step switching feature in the loop persists, indicating that the contribution of the magnetic moment from the substrate is negligible. Therefore, we believe that the two-step switching feature in the MH loop is due to the presence of multiple magnetic domains, consistent with prior reports on FGT films grown on Al_2O_3(0001) substrates.[3]

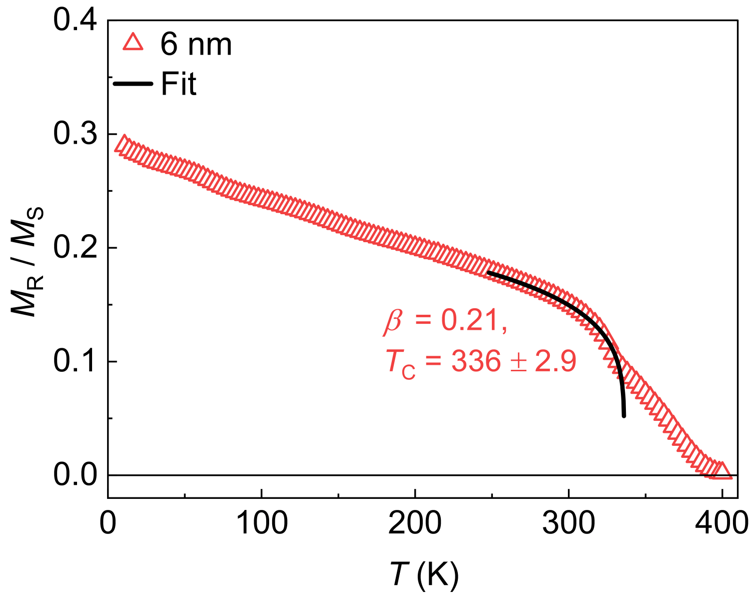

Figure S4: (a) Temperature dependence of remanent magnetization/saturation magnetization (M_R/M_S) for 6 nm FGaT film with the corresponding fit obtained using the equation, $T \propto (1 - T/T_C)^{\beta}$.

S4: $M_R/M_S - T$ Curve for 6 nm FGaT film.

We performed SQUID measurement on the 6 nm FGaT film and plotted the remanent/-saturation magnetization (M_R/M_S) as a function of temperature as shown in Fig. S4. We fit the curve with the equation: $T \propto (1 - T/T_C)^{\beta}$ and determined the value of $T_C = 336 \pm 2.9$ and $\beta = 0.21$. The value of $\beta = 0.21$ for the 6 nm FGaT film suggests that the system exhibits characteristics of 2D ferromagnetism[4–6]. However, a weak remanent magnetization with robust PMA persists up to 400 K, in the form of "tail-like" behavior, consistent to what has previously been observed for other 2D ferromagnets, such as $CrSiTe_3$[7], $CrCl_3$[8], $CrTe_2$[5,9], Fe_3GaTe_2[10], and Fe_3GeTe_2[6,11]. Therefore, the high-temperature magnetic stability of these 2D ferromagnetic films indicates the existence of finite PMA in the form of remanent magnetization, originating from the intrinsic layered structure of FGaT.

S5: XAS and XMCD sum rule analysis.

To extract the contribution of spin (μ_s) and orbital (μ_l) magnetic moments in the FGaT thin film, we performed sum-rule analysis on the XAS and XMCD spectra at the Fe-$L_{2,3}$ edges. In Fig. S5(a) & (b), the value of μ_l was obtained from the XMCD integral (I_m) over the L_3 edge of Fe, while the effective μ_s^{eff} was calculated from a combination of XMCD integral (I_m) and XAS integral (I_a) over both the L_2 and L_3 edges. To accurately determine the values of μ_s^{eff} and μ_l, we use the different absorption and dichroic integrals formulae below[1,12,13]:

$$\mu_s^{\text{eff}} = -2\langle S_\text{z}\rangle - 7\langle T_\text{z}\rangle = -\frac{N_\text{h}}{P\cos(\theta)}\frac{(6p-4q)}{r}, \tag{S1}$$

and

$$\mu_l = -\langle L_\text{z}\rangle = -\frac{N_\text{h}}{P\cos(\theta)}\frac{4q}{3r}, \tag{S2}$$

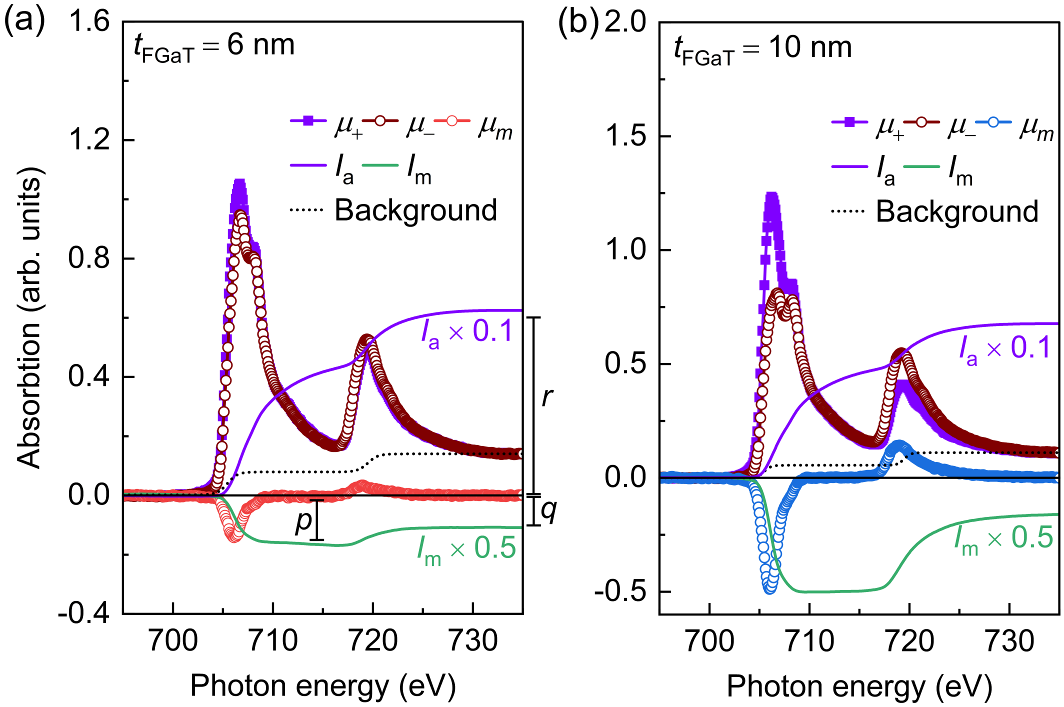

Figure S5: XAS, XMCD, integrals and the background spectra of Fe-edge, measured at 15 K, with the sum rule analysis for (a) 6 nm and (b) 10 nm thick FGaT/graphene heterostructure.

where,

$$p = \int_{L_3} (\mu_m), \; q = \int_{L_2+L_3} (\mu_m), \text{ and } r = \int_{L_2+L_3} \left[\frac{(\mu_+ + \mu_-)}{2} - Background\right]$$

Here, $\mu_m (= \mu_- - \mu_+)$ is the XMCD signal. The XMCD contrast is directly proportional to the Fe magnetic moment (in μ_B/atom) projected along the beam direction. The symbols μ_- and μ_+ refer to XAS spectra taken under $\pm H$ magnetic field. I_a and I_m are the integrated plots of XAS and XMCD plots. For both cases, the background signal is known to originate from the double step function. This background signal was already subtracted for the integrals I_a of the XAS plot as shown in Fig. S5(a) & (b). L_z, S_z, and T_z are the atomic orbital, spin magnetic moment, and magnetic dipole operator, respectively. N_h is the number of holes in $3d$ shell (for Fe in valence state of +2, the value 3.4 is used in sum-rule), P is the degree of circular polarization of X-ray beam (77% polarization of photon energy). Here, θ represents the angle between the external magnetic field (H), which is parallel to the beam direction and the film surface.

References

(1) Lv, H.; da Silva, A.; Figueroa, A. I.; Guillemard, C.; Aguirre, I. F.; Camosi, L.; Aballe, L.; Valvidares, M.; Valenzuela, S. O.; Schubert, J.; others Large-Area Synthesis of Ferromagnetic $Fe_{5-x}GeTe_2$/Graphene van der Waals Heterostructures with Curie Temperature above Room Temperature. Small **2023**, 19, 2302387.

(2) Kang, L.; Ye, C.; Zhao, X.; Zhou, X.; Hu, J.; Li, Q.; Liu, D.; Das, C. M.; Yang, J.; Hu, D.; others Phase-controllable growth of ultrathin 2D magnetic FeTe crystals. Nat. Commun. **2020**, 11, 3729.

(3) Liu, S.; Yuan, X.; Zou, Y.; Sheng, Y.; Huang, C.; Zhang, E.; Ling, J.; Liu, Y.; Wang, W.;

Zhang, C.; others Wafer-scale two-dimensional ferromagnetic Fe_3GeTe_2 thin films grown by molecular beam epitaxy. NPJ 2D Mater. Appl. **2017**, 1, 30.

(4) Gong, C.; Li, L.; Li, Z.; Ji, H.; Stern, A.; Xia, Y.; Cao, T.; Bao, W.; Wang, C.; Wang, Y.; others Discovery of intrinsic ferromagnetism in two-dimensional van der Waals crystals. Nature **2017**, 546, 265–269.

(5) Zhang, X.; Lu, Q.; Liu, W.; Niu, W.; Sun, J.; Cook, J.; Vaninger, M.; Miceli, P. F.; Singh, D. J.; Lian, S.-W.; others Room-temperature intrinsic ferromagnetism in epitaxial $CrTe_2$ ultrathin films. Nat. Commun. **2021**, 12, 2492.

(6) Park, S. Y. et al. Controlling the Magnetic Anisotropy of the van der Waals Ferromagnet Fe_3GeTe_2 through Hole Doping. Nano Lett. **2020**, 20, 95–100.

(7) Zhang, J.; Cai, X.; Xia, W.; Liang, A.; Huang, J.; Wang, C.; Yang, L.; Yuan, H.; Chen, Y.; Zhang, S.; others Unveiling electronic correlation and the ferromagnetic superexchange mechanism in the van der Waals crystal $CrSiTe_3$. Phys. Rev. Lett. **2019**, 123, 047203.

(8) McGuire, M. A.; Clark, G.; Kc, S.; Chance, W. M.; Jellison Jr, G. E.; Cooper, V. R.; Xu, X.; Sales, B. C. Magnetic behavior and spin-lattice coupling in cleavable van der Waals layered $CrCl_3$ crystals. Phys. Rev. Mater. **2017**, 1, 014001.

(9) Fujisawa, Y.; Pardo-Almanza, M.; Garland, J.; Yamagami, K.; Zhu, X.; Chen, X.; Araki, K.; Takeda, T.; Kobayashi, M.; Takeda, Y.; others Tailoring magnetism in self-intercalated $Cr_{1+\delta}Te_2$ epitaxial films. Phys. Rev. Mater. **2020**, 4, 114001.

(10) Wu, S. et al. Robust ferromagnetism in wafer-scale Fe_3GaTe_2 above room-temperature. Nat. Commun. **2024**, 15, 10765.

(11) May, A. F.; Calder, S.; Cantoni, C.; Cao, H.; McGuire, M. A. Magnetic structure and

phase stability of the van der Waals bonded ferromagnet $Fe_{3-x}GeTe_2$. Phys. Rev. B **2016**, 93, 014411.

(12) Chen, C. T.; Idzerda, Y. U.; Lin, H.-J.; Smith, N. V.; Meigs, G.; Chaban, E.; Ho, G. H.; Pellegrin, E.; Sette, F. Experimental Confirmation of the X-Ray Magnetic Circular Dichroism Sum Rules for Iron and Cobalt. Phys. Rev. Lett. **1995**, 75, 152–155.

(13) Backes, D.; Fujita, R.; Veiga, L.; Mayoh, D.; Wood, G.; Dhesi, S.; Balakrishnan, G.; van der Laan, G.; Hesjedal, T. Valence-state mixing and reduced magnetic moment in $Fe_{3-\delta}GeTe_2$ single crystals with varying Fe content probed by X-ray spectroscopy. Nanotechnol. **2024**, 35, 395709.
